# Functional Properties and Antimicrobial Activity from Lactic Acid Bacteria as Resources to Improve the Health and Welfare of Honey Bees

**DOI:** 10.3390/insects13030308

**Published:** 2022-03-21

**Authors:** Massimo Iorizzo, Francesco Letizia, Sonia Ganassi, Bruno Testa, Sonia Petrarca, Gianluca Albanese, Dalila Di Criscio, Antonio De Cristofaro

**Affiliations:** 1 Department of Agricultural, Environmental and Food Sciences (DiAAA), University of Molise, Via De Sanctis snc, 86100 Campobasso, Italy; iorizzo@unimol.it (M.I.); f.letizia@studenti.unimol.it (F.L.); bruno.testa@unimol.it (B.T.); sonia_petrarca@libero.it (S.P.); g.albanese@studenti.unimol.it (G.A.); d.dicriscio@unimol.it (D.D.C.); decrist@unimol.it (A.D.C.); 2 Conaproa, Consorzio Nazionale Produttori Apistici, 86100 Campobasso, Italy

**Keywords:** honey bee, gut microbiota, lactic acid bacteria, functional properties, antimicrobial activity

## Abstract

**Simple Summary:**

Honey bees play a pivotal role in the sustainability of ecosystems and biodiversity. Many factors including parasites, pathogens, pesticide residues, forage losses, and poor nutrition have been proposed to explain honey bee colony losses. Lactic acid bacteria (LAB) are normal inhabitants of the gastrointestinal tract of honey bees and their role has been consistently reported in the literature. In recent years, there have been numerous scientific evidence that the intestinal microbiota plays an essential role in honey bee health. Management strategies, based on supplementation of the gut microbiota with probiotics, may be important to increase stress tolerance and disease resistance. In this review, recent scientific advances on the use of LABs as microbial supplements in the diet of honey bees are summarized and discussed.

**Abstract:**

Honey bees (*Apis mellifera*) are agriculturally important pollinators. Over the past decades, significant losses of wild and domestic bees have been reported in many parts of the world. Several biotic and abiotic factors, such as change in land use over time, intensive land management, use of pesticides, climate change, beekeeper’s management practices, lack of forage (nectar and pollen), and infection by parasites and pathogens, negatively affect the honey bee’s well-being and survival. The gut microbiota is important for honey bee growth and development, immune function, protection against pathogen invasion; moreover, a well-balanced microbiota is fundamental to support honey bee health and vigor. In fact, the structure of the bee’s intestinal bacterial community can become an indicator of the honey bee’s health status. Lactic acid bacteria are normal inhabitants of the gastrointestinal tract of many insects, and their presence in the honey bee intestinal tract has been consistently reported in the literature. In the first section of this review, recent scientific advances in the use of LABs as probiotic supplements in the diet of honey bees are summarized and discussed. The second section discusses some of the mechanisms by which LABs carry out their antimicrobial activity against pathogens. Afterward, individual paragraphs are dedicated to Chalkbrood, American foulbrood, European foulbrood, Nosemosis, and Varroosis as well as to the potentiality of LABs for their biological control.

## 1. Introduction

Honey bees play a crucial role in the maintenance of wider biodiversity, ecosystem stability, and agricultural production through pollination [1,2,3]. While global stocks of managed honey bee colonies appear to be increasing, significant decline and colony losses of wild and domestic bees have been reported in many parts of the world [4,5,6,7].

Multiple biotic and abiotic factors are associated with the honey bee colony losses [4,8,9,10,11,12,13]. Furthermore, there is a growing consensus that parasites and pathogens are among the most significant threats to the management of bee colonies [14,15].

In-depth knowledge of these factors is essential and a prerequisite for developing measures to ensure both healthy bees and sustainable pollination. The aforementioned factors can also influence the honey bees’ gut microbiota. Its dysbiosis could weaken the honey bees, thus contributing to the phenomenon of Colony Collapse Disorder (CCD) [16,17,18]. Moreover, the gastrointestinal tract of honey bee’s larvae and adult bees is the infection and transmission site of different pathogens, such as *Ascosphaera apis*, *Nosema ceranae*, *Paenibacillus larvae*, *Melissococcus plutonius*, and viruses. These pathogens may cause economic losses in agriculture, affecting the survival of managed and wild honey bees [19,20,21,22]. Recently, many different control measures, such as fungicides, antibiotics, heterocyclic organic compounds (indoles), and bacteriophages, have been used to control honey bee diseases [23,24]. Most of these products were promising in terms of controlling the growth of pathogens both in vitro and in vivo. Nevertheless, these approaches could be useful as therapy, but are often ineffective for prophylactic purposes, leaving the honey bee colonies vulnerable to diseases. In addition, the use of antibiotics in beekeeping is legally banned in many countries of the European Union [25], due to the risks for both human and honey bee health [17,26], and to the uncontrolled spread of antimicrobial genes [27]. Therefore, there is a growing interest in new effective means of controlling disease and improvement in honey bee health, as well as providing benefits for agriculture by increasing yield and quality of crop production. The use of naturally occurring compounds for disease control could be an interesting approach that needs to be further investigated because the findings to date have not always been of biological relevance [28,29,30].

The gut microbiota is fundamental for honey bee’s growth and development, immune function, protection against pathogen invasion [17]. A well-balanced microbiota is essential to support honey bee health and vigor, moreover, the structure of the intestinal bacterial community can become an indicator of the honey bee health [31,32,33].

The gut microbial communities can also provide an important new tool to improve disease management strategies and contribute to the development of novel and sustainable disease monitoring approaches [34,35,36]. More in general, the manipulation and the exploitation of the insect microbiota could be effective in developing strategies for the management of insect-related problems [37,38]. Indeed, this approach, generally defined as ‘Microbial Resource Management’ (MRM), was described as ‘Symbiont Resource Management’ (SMR) when applied to symbiotic microorganisms. The MRM refers to the proper management of the microbial resource, present in a given ecosystem, in order to solve practical problems through the use of microorganisms. One of the environmental hot topics is represented by the gastrointestinal tract (GIT) defined as an “outside world within living animals” [39]. The main objective is to control and steer microbial communities, and microbial processes, in the most sustainable way.

Moreover, the protection against pathogens and/or parasites is one of the frequently associated aspects of a balanced intestinal microbiota. Indeed, it is widely known that the early stages of pathogens infection can be eased by any nutritional or environmental stress causing microbial dysbiosis. In several studies, it has been proven that, among the microbial symbionts associated with the honey bee, the lactic acid bacteria have a probiotic effect on bees by stimulating their immunity and helping them to overcome pathogen attacks [40,41,42,43]. Different mechanisms, among which the direct pathogen inhibition by the release of antimicrobial compounds, the stimulation of the immune system, and the competitive exclusion, mediated by the microbial symbionts, could be involved in the honey bee protection.

In this review, we briefly introduce the presence and the role of the LAB community in the honey bees’ gut microbiota and, subsequently, we discuss the utilization of LABs as probiotics and their potential for the biocontrol of some honey bee diseases.

## 2. Gut Microbiota of Honey Bee: Presence and Role of Lactic Acid Bacteria (LAB)

The GIT microbiota structure of honey bees (*Apis mellifera*) is both unique and highly specialized; in detail, the dominant bacterial phyla belong to *Proteobacteria*, *Actinobacteria*, *Bacteroidetes,* and *Firmicutes* [44,45]. In social insects, such as the honey bees, the intestinal bacteria are transmitted and shared by colony members through oral–fecal and trophallactic transmission. However, consumption of stored pollen or bee bread, contact with older bees within the hive, and hive material during the adult phase are also involved in transmitting and sharing the bacteria [17,44,45].

The lactic acid bacteria (LAB) are normal inhabitants of the GIT of many insects, and their presence in the honey bee intestinal tract has been consistently reported in the literature [46,47,48]. These bacteria belong to a biologically defined group where lactic acid is produced during homo- or hetero-fermentative metabolism.

Soil and plants are considered the hypothetical first niche of the ancestral LAB, followed by the gut of herbivorous animals [49]; the transition from the soil and the plants to the animal gut occurred by three areas of genomic adaptation [50,51]: resistance to host barriers, adhesion to intestinal cells, and fermentation.

A bees’ gut is the optimal microenvironment for LAB as it is defined by microaerobic conditions, presence of nectar and sugars, optimal temperature. Olofsson et al. [52] suggested that bees and their microbiota are mutually dependent, in fact, LABs received a niche in which nutrients were available and bees gained protection [35,53,54].

LABs’ importance is further emphasized by their ecological distribution, which is not limited to adult bee gut only, in fact, they have been isolated from larval guts [55] and the honey stomach of adult bees [56]. This latter structure, adjacent to the midgut, is a further relevant microbial niche associated with food storage and liquid transfer (water, nectar, and royal jelly). In addition, LABs are also dominant in the hive environment (beebread, honey, wax, and comb) [46,47,48,52,53,54,56,57,58].

LABs have also been extensively studied in animals and humans because of their probiotic properties, which have led to their well-built commercial exploitation in the food, feed, and pharmaceutical market [59,60,61,62,63]. The finding that a component of the honey bee gut microbiota was represented by LABs has increased the interest of scientists in looking for similarities and analogies with the probiotic bacteria widely investigated in humans and animals.

## 3. Functional Properties of LAB

There are several properties through which LABs can provide a specific health benefit for the honey bees [60,61,62].

The following section discusses some functional properties of LAB supplemented as probiotics in bee feeding. Table 1 provides an overview of the main results obtained in several studies.

### 3.1. Heavy Metals Detoxification

Global environmental pollution is increasing because of continuous and intense industrial and technological development, in particular, toxic metals, such as heavy metals (HM), are considered one of the major harmful pollutants [81,82,83,84]. HMs, once found in soil or water, are not easily eliminated; they cause irreversible damage to the survival, feeding, growth, and impair the behavior of the organisms, including honey bees [85,86,87,88,89]. It has been widely demonstrated that the honey bee populations are susceptible to several environmental threats, including HMs [81]. The honey bees can be exposed to HMs when foraging on contaminated honey and pollen resources and, in some cases, by airborne exposure [90,91]. The HMs also bioaccumulate in larval and adult stages, in the colony’s honey, wax, and propolis supplies [86,91], making the honey bees excellent bioindicators of HMs presence in the environment [92,93]. Some reports indicate that the honey bee cell ultrastructure can be adversely affected by HMs, inducing cell apoptosis that can disrupt cell vigor and cell proliferation. HMs can also negatively affect the genetic material, resulting in mutation, and in addition, they also cause neurotoxic effects [86,89,91,94,95,96,97,98,99,100]. Other studies have shown that the HMs may affect antioxidant capacity and immunocompetence in honey bees [87,88,101].

Many bacteria, including the LABs, appear to have the ability to efficiently remove the HMs through two mechanisms: biosorption and bioaccumulation [102,103,104,105].

Biosorption refers to the binding of metals onto the cell wall’s surface and it is a simple physicochemical process, whereas the bioaccumulation process refers to the intracellular accumulation of metals that occur in two stages, biosorption and bioaccumulation by transporting the metals across the cell wall and membrane [106,107,108]. Recently, the next generation of probiotics has attracted increasing attention [103,109,110] for their ability to alleviate HMs toxicity, although, most of the studies have been performed with an in vitro digestion or animal model. [75,103,111,112,113,114,115,116,117,118]. Based on this research, specific LABs could be used as a new dietary therapeutic strategy against HMs toxicity. In this regard, Rothman et al. [85] demonstrated that some honey bee symbiotic LABs are capable of in vitro metals’ bioaccumulation. However, these results are preliminary and so, more in-depth, systematic, and epidemiological studies need to be performed on honey bees.

### 3.2. Mitigation of Pesticides Effect

Pesticides, such as insecticides and fungicides, are considered one of the possible stressors causing the general decline in honey bees and colony losses [5,12,119]. The exposure of honey bees to pesticides also causes microbial dysbiosis and immunosuppression, rendering them more susceptible to pathogens; furthermore, the interactions between pesticides and pathogens may exacerbate honey bees’ mortality [120,121,122,123,124,125,126,127,128,129,130]. A novel concept may be the administration of lactic acid bacteria to mitigate the harmful effects of pesticides. There are several mechanisms through which the treatment with probiotics could act on pesticide intoxication; for example, the treatment with *Pediococcus acidilactici* restored the expression of two genes, which were altered by pesticide co-exposure, coding for serine protease 40 and vitellogenin [131]. Moreover, the benefit of LAB supplementation is a reduction in pesticide uptake through their degradation [132,133,134,135] or sequestration of ingested organophosphate pesticides, which has been associated with reduced intestinal absorption and insect toxicity with appropriate models [136,137]. In other model organisms, LABs have been shown to reduce toxicity and exert a protective effect on the host [135,138,139,140], thus establishing a basis for future studies to investigate this potential in honey bees. Recently, some authors have highlighted how the resistance and capacity of LABs for degrading organophosphorus pesticides is strain-dependent [134,141] and showed the feasibility of the LAB to be developed into probiotic products capable of alleviating oxidative damage caused by pesticides in vivo [142].

Based on this knowledge, probiotic supplementation with appropriate LAB cultures could mitigate the sublethal effects of pesticides by reducing pesticide uptake, improving pathogen resistance, and mitigating sublethal effects on colony development. Until chemical agents are no longer used in agriculture, the ability to supplement honey bees with probiotics could help the insects to fight the unintended pernicious effects [143].

### 3.3. Adhesion to Intestinal Mucosa and Enhancement of the Epithelial Barrier

Adhesion to intestinal epithelial cells is a prerequisite for the colonization of probiotic bacteria, leading to transitory colonization that would foster the immune response and, at the same time, stimulate the intestinal barrier and metabolic functions. In addition, this ability to adhere to the host may serve a protective role against undesirable microorganisms through competition for host cell-binding sites [108,144,145,146].

As reported in a number of studies, during this adaptation phase, bacteria produce extracellular polymeric substances (EPS), containing biological macromolecules, some of which (polysaccharides, proteins, nucleic acids, and lipids) are also responsible for the cohesion of microorganisms and are implicated in the production of biofilms [146,147].

### 3.4. Participation in the Digestive Process

The honey bee gut microbiota, as well as that of other insects, synthesize essential nutritional compounds and improve the digestion efficiency and availability of nutrients [17,47,148]. A properly functioning gut microbiota is closely connected to the health of the honey bee since it provides countless enzymatic activities to break down the complex sugars of the honey bees’ diet. Iorizzo et al. [48] proved that some *Lactiplantibacillus plantarum* (previously *Lactobacillus plantarum*) strains isolated from honey bee gut possess both alpha- and beta-glycosidase activities. The enzyme beta-glycosidase in association with other enzymes, cellulase, and hemicellulase produced by bee intestinal symbionts, such as Gilliamella, contributes to the hydrolysis of cellulose [149]. The alpha-glycosidase converts maltose to glucose and with alpha-amylase, is involved in the starch breakdown [150].

Honey bees collect food rich in carbohydrates, such as sucrose, glucose, and fructose, which are important for the development and well-being of their colonies [151]. However, other carbohydrates present in their diet in lesser quantities, such as monosaccharides (e.g., mannose, galactose, xylose, arabinose, rhamnose) and oligosaccharides (e.g., lactose, melibiose, raffinose, and melezitose), may be toxic to bees as they do not have specific enzymatic activity for their metabolization [152,153]. Iorizzo et al. [154,155] evidenced that some *Apilactibacillus kunkeei* (previously *Lactobacillus kunkeei*) and *Lp. plantarum* strains can metabolize arabinose, galactose, lactose, mannose, melibiose, melezitose, and raffinose. As they are able to simultaneously intervene in the breakdown of complex polysaccharides and metabolize toxic sugars, the role of LABs in enhancing food tolerance and maintaining the health of their hosts could be considerable [156].

### 3.5. Antioxidative Activity

Recent research demonstrated that several biotic and abiotic factors, induce oxidative stress and impair the antioxidant defensive capacity of honey bee larvae [9,157,158,159,160].

Oxidative stress is an important process that can cause severe negative effects in eukaryotic organisms. Reactive oxygen species (ROS) are produced during normal metabolic processes and are responsible for oxidative stress. To prevent or reduce ROS-induced oxidative stress, insects use various enzymatic mechanisms that cause oxidative inactivation (superoxide dismutase, catalase, and peroxidase) or removal of ROS at the intracellular level through the enzymes glutathione peroxidase (GPX) and glutathione reductase (GSR) [161,162,163].

These particular enzyme activities are relevant for the health of honey bees when they are under biotic and abiotic stressors, such as nutritional and thermal stress, parasites, heavy metals, and/or pesticides [9,129,157,164,165,166,167,168]. Oxidative stress can also be a consequence of some honey bee diseases; in fact, during the excessive growth of pathogens, the levels of ROS in the infection site increase [160]. Dussaubat et al. [169], and more recently Li et al. [158], reported that the oxidative stress in honey bee larvae and the decreased levels of metabolites involved in mitigating oxidative stress induced by *Ascosphera* apis could disrupt the antioxidant defenses of the infected larvae. Antioxidant enzymatic activity and the amounts of certain metabolites (e.g., taurine, docosahexaenoic acid, and L-carnitine) involved in reducing oxidative stress were significantly decreased in the gut of infected larvae [158]. In recent years, particular attention has been focused on the application of LABs as natural antioxidants. Some strains belonging to this group have both enzymatic and non-enzymatic antioxidant activity, which can reduce the oxidative damage caused by the accumulation of ROS during the digestive process [170,171,172]. Probiotic LABs have complex antioxidant mechanisms, and different strains use different mechanisms: chelation of toxic ions (Fe^2+^ and Cu^2+^); synthesis of antioxidant compounds (e.g., glutathione, butyrate, folate, and exopolysaccharides); activation of transcription of enzymes that neutralize free radicals [173,174,175]. Further research aimed at the selection, and diet utilization, of appropriate probiotics that can contribute to the reduction in oxidative stress in honey bees, would be interesting.

## 4. Antimicrobial Activity of LAB

The first part of this section discusses some of the mechanisms by which LABs carry out their antimicrobial activity against pathogens. Afterward, individual paragraphs are dedicated to some main diseases and the potentiality of LABs for their biological control. Table 2 provides an overview of the main results obtained in recent years.

### 4.1. Immune System Stimulation

Several abiotic and biotic factors, which include pathogens (parasites, fungi, bacteria, and viruses), the alteration or loss of ecosystems, and/or the use of agrochemicals, are contributing to the decline in bee populations. All these factors alter the defense mechanisms of the bee immune system [33,140,203,204,205,206,207] which includes physical barriers and generalized cellular and humoral responses to infectious and parasitic organisms [208,209]. The humoral response is the most important defense system in the honey bee and is mediated by the production of antimicrobial peptides (AMPs), such as apidaecin, abaecin, hymenoptaecin, and defensin [210]. These compounds act by restricting the enzymes necessary for pathogen growth and duplication, forming channels in the pathogens’ cell membranes [211]. Probiotic bacteria can exert an immunomodulatory effect, in particular *Lactobacilli* and *Bifidobacteria*, but also the gamma-proteobacterium *Frischella perrara* have shown to enhance the honey bee immune system [76,212,213]. Yoshiyama et al. [80], using a mixture of probiotic bacteria strains, (one strain of *Enterococcus thailandicus*, three of *Weissella cibaria*, one of *W. viridescens,* and two of *Lactobacillus curvatus*), achieved a significant upregulation in abaecin, defensin, and hymenoptaecin peptides, both in adult and larvae bees. The combination of *Bifidobacterium longum, B. breve, B. infantis, Lactobacillus rhamnosus, L. reuteri, L. acidophilus, Lp. plantarum*, and *L. casei* caused the AMPs upregulation, which boosted the honey bee immune system [76]. In addition, *B. asteroides* and *Fructobacillus pseudoficulneus* strains, isolated from honey bee worker gut and bee bread, exerted a considerable upregulation in apidaecin synthesis, and the effects on the honey bee immune system are strain-specific [78]. Moreover, a pollen-based probiotic preparation improved immunity in bee colonies, and thus increased their resistance to infectious diseases and stressful conditions [74].

### 4.2. Competitive Exclusion of Pathogenic Microorganisms

Many honey bee pathogens need to adhere to the mucosal layer of the gastrointestinal tract to start the infectious process. By colonizing the intestine and adhering to its epithelial surface, bacteria interfere with the adhesion of pathogens. [154]. This property is also known as “competitive exclusion” [214]. The existence of any microbial population depends on its ability to compete for chemicals and available energy with other microbes in the same environment. Some probiotic LABs consume nutrients or sequester chemicals that are essential for the growth of pathogens. For example, siderophores are low-molecular-weight ferric iron-chelating agents that are able to dissolve precipitated iron, or extract it from iron complexes, to make it available for bacterial growth [214,215].

### 4.3. Production of Antimicrobial Substances

Probiotic bacteria produce bactericidal or bacteriostatic substances, including bacteriocins, hydrogen peroxide, siderophores, lysozymes, proteases, which act on other microbial populations [214,216,217,218,219,220]. In addition, some bacteria produce organic acids, and volatile fatty acids (e.g., lactic, acetic, butyric, and propionic acids), that can result in the reduction in pH in the gastrointestinal lumen, thus preventing the growth of opportunistic pathogenic microorganisms [61,216]. The processes behind the inhibition activity may include a number of compounds that can lead to membrane disruption (fatty acids or peptides), H^+^ gradient interference, or enzyme inhibition. In addition, the LAB antimicrobial action is often due to a complex interaction among different compounds [155,217]. Organic acids, particularly acetic acid and lactic acid, have a strong inhibitory effect against Gram-negative bacteria, and they have been considered the main antimicrobial compounds responsible for the probiotic inhibitory activity against pathogens. The undissociated form of the organic acid enters the bacterial cell and dissociates inside the cytoplasm. The eventual lowering of the intracellular pH or the intracellular accumulation of the organic acid ionized form can lead to pathogen death [218]. In addition, many LABs produce antibacterial peptides, including bacteriocins and small AMPs, whose common mechanisms of bacteriocin-mediated killing include the destruction of target cells by pore formation and/or inhibition of cell wall synthesis [219]. Bacteriocins exhibit antimicrobial activity with a variable spectrum depending on the peptide, which may target several bacteria [221,222,223]. Moreover, there is scientific evidence that bacteriocins are effective against some bacterial infections of honey bees [224]. Some studies, which will be cited later, conducted on the antimicrobial activity of probiotic LAB (Table 2), have not shown biological relevance due to the partial inhibition of pathogens. However, it should be noted that the benefit of probiotics is the result of a sum of actions (antimicrobial, detoxifiers, antioxidants, and immunostimulants) that overall can improve the overall health and well-being of bees.

### 4.4. Chalkbrood

Chalkbrood is a fungal honey bee brood disease caused by *Ascosphaera apis* [225,226], a heterothallic parasitic fungus, belonging to the phylum Ascomycota. This disease is currently widespread throughout the world and different studies have pointed out that chalkbrood incidence may be increasing [225,227]. This economically important disease, although it does not destroy the entire colony, weakens it, and can lead to significant losses of both honey bees and colony productivity [228,229,230]. In addition, *A. apis* infection, inducing oxidative stress in honey bee larvae, may compromise their antioxidant defensive capacity [158].

Over the years, several chemotherapeutic compounds and strategies have been tested and developed to control the chalkbrood disease [231,232,233,234,235,236], but to date, there are no effective compounds to properly control the disease. Moreover, chemotherapeutic compounds are often expensive and release residues in honey bee products, representing a risk for human health [237], as well as causing an imbalance in the normal honey bee intestinal microbiota [238]. Therefore, a growing interest has been reported in developing chalkbrood-controlling strategies. Among natural compounds, several essential oils demonstrated a significant antifungal activity [239,240,241,242]. Propolis exhibits inhibiting activities against pathogens, including *A. apis*, and in particular, it has been observed that the effects depend on the plant resources of each region from which the propolis is produced [243,244,245,246].

In recent years, various studies have also led to the hypothesis that the honey bee intestinal microbial community may represent a future alternative strategy for the control of the chalkbrood disease. *Bacillus subtilis*, *B. megaterium*, and *B. circulans* [247] have shown in vitro inhibition activity against *A. apis.* Sabaté et al. [248] in a study on the inhibition of *P. larvae* and *A. apis* by *Bacillus subtilis*, hypothesized that *B. subtilis* secreted antimycotic compounds, and more recently Omar et al. [249] demonstrated an antagonistic activity of *B. subtilis* and *Pseudomonas fluorescens* strains, isolated from the gut of *Apis mellifera carnica*, against *A. apis.* Tejerina et al. [176] demonstrated that the in vitro application of *L. melliventris* LSAM, *L. helsingborgensis* LSAI, and *A. kunkeei* LSAJ strains, isolated from beebread, affected the growth and sporulation of *A. apis.* The same authors have shown that feeding honey bee larvae with LAB (*L. melliventris*, *L. helsingborgensis*, and *A. kunkeei*) in sugar syrup over 5 months, reduced larval mummification in chalkbrood disease by over 80% [176]. Iorizzo et al. [154,178] proved that *L. kunkeei* K7, K18, K34, K40, K41, K45, K55, K64, and K112 (current name *Apilactobacillus kunkeei*) and *Lactiplantibacillus plantarum* P8, P25, P86, P95, and P100 not only inhibited *A. apis* but were also suitable in the preparation of a “probiotic syrup” to reinstate the symbiotic communities of the honey bee intestine in case of dysbiosis and to perform a prophylactic action against *A. apis.*

### 4.5. American Foulbrood (AFB) and European Foulbrood (EFB)

*Paenibacillus larvae* (Bacillales, Paenibacillaceae), a flagellated, Gram-positive bacterium, is the causative agent of the quarantine disease American foulbrood (AFB), the most severe and cosmopolitan brood disease affecting *A. mellifera* larvae and pupae [14,15,21]. The infection follows the ingestion of spore-contaminated food provided to the larvae by honey bee nurses [250]. The spores germinate in the midgut lumen, where the vegetative bacteria massively proliferate, and secrete secondary metabolites, to counter microbial competitors, a chitin-degrading enzyme, responsible for the peritrophic matrix degradation [251,252,253], and other enzymes that allow the breaching of the midgut epithelium. The vegetative bacteria invade the hemocoel [254], causing the death of the larvae several days after infection, turning them into a ropy mass that dries and becomes a continuous source of infection, releasing a large number of bacterial spores [250]. These spores infect and kill a great number of larvae, resulting in the lack of offspring that leads to the collapse of the entire colony, causing considerable economic loss to beekeepers [21,255]. The infectious spores can spread within the colonies by nurse bees performing inhive tasks, such as cleaning, and through the feeding of larvae with spore-contaminated food [256]. The spore transmission can also occur between colonies through swarming, robbing, and drifting bees, as well as by beekeeping practices, e.g., through the movement of contaminated hive materials, such as honey or equipment [257,258,259]. *P. larvae* can produce very resilient spores which can remain viable for decades [260]. *Melissococcus plutonius* (Lactobacillales, Enterococcaceae) is a Gram-positive bacterium [261] that causes epidemic outbreaks of European foulbrood (EFB), one of the most worldwide detrimental brood diseases affecting honey bees, such as *Apis mellifera* L. [20,262]. Several other bacteria, typically found in the hive environment, may be associated with EFB disease, although the role of the secondary invaders in disease development is not yet entirely clear [20,55,263]. Honey bee larvae can be infected by ingestion of contaminated food, administered by adults [20], and once *M. plutonius* reach the intestinal tract, it quickly multiplies and can deprive the host of nutrients [264]. Diseased larvae are characterized by a typical change from white to a yellowish color, a foul or sour smell, they become flaccid, usually do not reach or complete the pupation stage and die 4–5 days after infection [20,265]. However, some infected larvae can survive and deposit the bacteria along with their feces in the comb, and they can survive long periods inside the hive [266]. *M. plutonius* in diseased colonies can be easily spread between colonies/apiaries [267,268].

The severe losses of brood, colony collapse, and extreme contagiousness make EFB and AFB economically important diseases, notifiable in many countries [15,269]. In most European countries, at least, the only measure to counteract them is the destruction of symptomatic colonies and the monitoring of neighboring apiaries to avoid pathogen spread [14,20,270]. However, strategies to treat and control the diseases vary across the world. In several countries, including the U.S. and Canada, antibiotics are commonly used for the treatment of infected colonies [15,271,272].

The use of antibiotics to control both diseases is an unsustainable strategy since they only affect vegetative form. The long-term treatment and their common use in prophylaxis, besides not eliminating the bacterial spores, has led to the development of antibiotic resistance [272,273,274,275,276]. Antibiotics also cause honey bee microbiota dysregulation [26], compromising the honey bee’s overall health status [17,18,31,268,277,278]; moreover, they may leave residues in honey [279,280]. For all these reasons, antibiotics are now strictly regulated or restricted in most European countries [279,280,281]. A recent study has investigated the potential of insect antimicrobial peptides for the development of a new class of insect-derived antibiotics to overcome AFB resistance to conventional antibiotics. The data obtained revealed a strong immune response against *P. larvae* in *P. larvae*-injected third instars of *A. m. jemenitica* and the presence of immune peptides in their plasma. Furthermore, the immune peptide fractions exerted significant in vivo therapeutic effects on *P. larvae*-infected first instars [282].

The application of alternative, natural strategies for EFB and AFB control could represent a suitable management approach [250,283]. Plant and propolis extracts, herbs, spices, and essential oils exhibit antimicrobial activity against *P. larvae* and *M. plutonius*, but, most of them have only been tested in vitro [250,284,285,286,287,288,289,290]. Macelignan and corosolic acid, the latter extracted from the banaba (*Lagerstroemia speciosa*) leaf, showed strong in vitro antibacterial activity against both *P. larvae* and *M. plutonius* [289]. Several studies are showing the in vitro ability of propolis extracts, from different botanical origins, or their constituents, to inhibit the growth of *P. larvae* [245,284,291,292,293]. Borba and Spivak [294], using colonies with and without a propolis envelope, demonstrated that the presence of the envelope provides an antimicrobial layer around the colony that protects the brood from *P. larvae* infection, resulting in a lower colony-level infection load. Some essential oils were tested in in vitro assays against *P. larvae* and *M. plutonius* [240,286,287,290,295,296]. The systemic administration of *Cinnamomum zeylanicum* EO to artificially infected nuclei of honey bee colonies prevented and controlled AFB [297,298]. The utilization of a favorable bacteria-based strategy for the prevention and biocontrol of honey bee pathogenic microorganisms offers interesting perspectives [42,43,131]. Different isolates of the bacteriocin-producing *Enterococcus*, often isolated from freshly collected pollen granules, showed strong in vitro inhibitory activity against *P. larvae* [188,189,190,191]. Yoshiyama et al. [80] demonstrated that nine LAB strains, belonging to *Enterococcus* sp., *Weissella* sp., and *Lactobacillus* sp., showed strong in vitro antagonistic activity against *P. larvae*, soon after oral administration, they were able to stimulate the honey bee innate immune response in vivo, which may be useful for preventing bacterial diseases in honey bees. A number of investigations also demonstrated that different strains belonging to LABs, among them *A. kunkeei* and *Lp. plantarum*, could be useful against *P. larvae*, inhibiting its survival and reducing pathogen load [177,180,181,183,185,186,187]. More recently, Iorizzo et al. [155] demonstrated that *Lactiplantibacillus plantarum* P8, P25, P86, P95, and P100 exhibited in vitro antagonistic activity against *P. larvae*, showing, at the same time, suitable physical and biochemical characteristics for their use as probiotics in the honey bee diet. In vivo studies demonstrated that the addition of gut bacteria to the diet significantly reduced the mortality of the infected *A. m. jemenitica* larvae [209] and that a diet supplemented with lactobacilli improved *A. mellifera* survival and hive resilience against *P. larvae* [214].

An in vitro study by Vasquez et al. [192] reported that *A. kunkeei* FF30-6, isolated from honey bees, showed antagonistic effects against *M. plutonius*, and this activity may be due to the production of anti-*M. plutonius* peptides or proteins [46,193]. Killer et al. [182] proved that *Lactobacillus apis* R4B, isolated from the stomach of honey bees, exhibits in vitro antagonistic effects against both *P. larvae* and *M. plutonius*. Another recent study has shown that the *A. kunkeei* V18 strain exhibit a strong in vitro inhibition for both *P. larvae* and *M. plutonius* [177]. Bacteria isolated from the gut of *Apis cerana japonica* F. have been shown to inhibit in vitro the growth of *P. larvae* [299]; while in vivo feeding bioassays proved that an isolate belonging to the genus *Bacillus* exhibited inhibitory activity against *M. plutonius* [36].

### 4.6. Nosemosis

*Nosema apis* (Zander 1909) and *N. ceranae*, [300] belonging to Microsporidia, obligate intracellular and spore-forming parasites, classified as fungi [301,302]. Both these species are etiologic agents of nosemosis, one of the most widespread and serious diseases of the adult honey bee [22,303,304,305]. *N. apis* is responsible for nosemosis type A [306], a disease that increases bee mortality in winter and causes a slow build-up in spring, makes weak and crawling bees, and reduces honey yield [307]. The disease also altered the infected worker bees’ flight behavior, reducing foraging ranges of colonies, and compromising their ability to provide pollination services [308]. *N. ceranae* provokes the nosemosis type C [306] that includes a wide range of effects on honey bees’ physiology and behavior, causes weakness, increases in colony mortality, and decreases in honey production; all factors that can contribute to colony collapse [309,310,311,312,313,314,315,316]. Moreover, *N. ceranae* infection can affect the host’s immune response leading to immune system suppression [204,206]. The acquisition of *Nosema* occurs via the fecal–oral pathway through the spores passed out of the host in excrement, which can contaminate the nesting environment, comb, floral resources, collected pollen, and water [22,307,310,317].

Because of the serious consequences of *Nosema* infections, there is a strong demand for the management of these pathogens. The antifungal fumagillin, used against nosemosis for several decades, has been banned by many countries (including the European Union) [318] due to its genotoxic and tumorigenic properties towards humans and toxicity to bees [319,320]. Therefore, the identification of alternative treatments is fundamental for honey bee health. Currently, several compounds, such as formic acid, are used for the management of *Nosema* infections [321]. Thymol and resveratrol were also reported to inhibit *N. cerane* [322,323,324]. Oxalic acid and Api-Bioxal^®^, a formulation based on dihydrate oxalic acid, were demonstrated to be active against *N. ceranae*, both in the laboratory and field [325,326,327]. The biological activity of other natural compounds towards *Nosema* infections has also been extensively explored; among them, essential oils and other organic extracts were reported to have anti-*Nosema* activity [328,329,330,331,332,333].

It is well known that midgut microorganisms have a part to play in the health of honeybees, and dietary integration with prebiotics and probiotics may offer a way to counteract *Nosema* infections. However, studies revealed that the administration of prebiotics or probiotics to honey bees has provided contrasting results. Chitosan, a prebiotic, increased honey bee resistance to *N. apis* infections [334], reduced *N. ceranae* spore load, and increased survivorship of the infected bees [335]. The administration of *Brassicaceae* defatted seed meals, as the integration of infected *A. mellifera* workers diet, leads to the inhibition of *N. ceranae*, and potential nutraceutical benefits were involved in the bee lifespan [336,337]. Some nutraceuticals, such as naringenin and carvacrol, and immuno-stimulatory compounds, administered in sugar syrup, reduced both spore multiplication and mortality during *N. ceranae* infection in honey bees [338]. Microbial supplements of the honey bee diet may have positive impacts on bee health and control nosemosis. Feeding the honey bee with the endogenous gut bacteria *Bacillus subtilis* reduced *Nosema* disease [248], and a surfactin, synthesized by the bacterium, was also proved to reduce *N. ceranae* development, acting either by direct exposure to purified spores or incorporated into the digestive tract of the bee [196]. Oral administration of organic acids, produced by *L. johnsonii* CRL1647, supplemented in syrup [67], provoked a *Nosema ceranae* occurrence reduction [66]. *Parasaccharibacter apium* improves honey bee resistance to *Nosema*, as well as the honey bee gut bacterial strains *P. apium* (*PC1* sp.) and Bacillus sp. (PC2 sp.); moreover, the commercial probiotics Bactocell^®^ R and Levucell SB^®^ have increased the survival of *Nosema* infected bees. However, these last four probiotics do not exert a direct antagonistic effect on *N. cerane* development [200,339]. A *Pediococcus acidilactici* strain (PA CNCM MA18/5 M) was found to be the most efficient probiotic against *N. ceranae*, significantly improving the infected honey bee lifespan [131]. The commercial probiotic Vetafarm (*L. acidophilus, L. delbruekii sub. bulgaricus, Lp. plantarum, L. rhamnosus, Bifidobacterium bifidum, Enterococcus faecium*), besides reducing *N. ceranae* proliferation, increases the survival of infected honey bees [198]. Conversely, the bacteriocins by *E. faecium* CRL1385 and *E. avium* DSMZ 17,511 did not exert any activity towards *N. ceranae* [196]. *Al. kunkeei* and *L. salivarius* A3iob administered to honey bee colonies reduced *Nosema* disease [77]. Gajger et al. [77] proved that administering the commercial probiotic EM^®^ PROBIOTIC FOR BEES was followed by a significant reduction in *Nosema* spp. spore counts in colonies and colonies’ strength were increased. On the contrary, the supplementation of the honey bees’ diet with the probiotic *Lactobacillus rhamnosus* and the prebiotic inulin showed no beneficial effect on the survival rates of honey bees infected with *N. ceranae* [199]. A significant increase in *Nosema* spore counts, compared to the *A. mellifera* infected control, was caused by a probiotic composed of *L. casei*, *Lp. plantarum*, *Saccharomyces cerevisiae*, and *Rhodopseudomonas palustris* [197,340]. RNA interference technologies, applied to both *Nosema* and honey bee, are also explored for *Nosema* disease control [301,302,303,304,305].

### 4.7. Varroosis

*Varroa destructor* is the main mite that can attack the honey bee (*Apis mellifera* L.). This parasite nourishes on the hemolymph of the honey bee and within a few years causes the colony to collapse [341,342]. *Varroa* mites can also host many pathogens, viruses, including Deformed wing virus (DWV) [343,344], and harmful bacteria [345]. Although in recent studies Ramsey et al. [342,346] proposed that *V. destructor* feeds on bee fat body, the caused damages always involve a reduction in protein content and hemocytes [347], leading to an imbalanced gut microbiota [348,349], and a decrease in host’s immune response [207,350,351]. Acaricides, such as fluvalinate, flumethrin, and amitraz, have successfully been used to control varroosis [352]. The use of these chemical compounds, however, has resulted in the development of resistant mites and the risk of acaricide residues in bee products, such as wax and honey. [353]. Therefore, there is an urgent need to find eco-friendly strategies that are both affordable and safe for humans and honey bees to control *Varroa* mites. Numerous studies have been conducted on natural remedies, such as plant extracts and essential oils, against *V. destructor* in honey bee colonies [354,355,356]. The efficacy of certain plant-based essential oils, such as eucalyptus, thyme, neem, sage, and grapefruit, against *Varroa* mites has been reported in many studies. [357,358,359,360]. However, the effectiveness of these methods is not constant and always effective, and the search for new strategies in Varroosis biocontrol is still ongoing [30,361]. Recent studies have highlighted the potential of LABs in the biocontrol of this disease. Tejerina et al. [194] demonstrated that *L. salivarius* A3iob reduced the levels of in situ varroosis by between 50 and 80%. *L. johnsonii* AJ5 and *E. faecium* *SM21* strains enhanced bee survival and increased bee proteins [201]. Sacca and Lodesani [202] demonstrated in vitro that bacterial cultures of *Al. kunkeei* BO-G12 caused 95–100% mortality of the mite in 3 days.

## 5. Conclusions

A variety of adverse factors, among which pests and pathogens are the major candidates, but also pesticide exposure, diet quantity, quality, and diversity as well as unfavorable weather and fodder conditions, contribute to the decline in honey bees. In addition, the beekeeper’s management practices strongly influence the health of honey bee colonies. All these factors, acting alone or in combination, affect honey bee colonies, causing possible serious disturbances in the composition of the honey bee microbiota.

Moreover, honey bees, like all living things, are constantly in contact with many microorganisms that can exert an impact on their health and well-being.

Several researchers have investigated the use of the gut microbial symbionts as a supplement in the honey bee diet and the results have shown that such microorganisms (multiple strains or single strain), or their metabolites, could improve the health status of the honey bees. The honey bee symbionts could be exploited to actively counteract bee pathogens and parasites, or to enhance immunity, and thus indirectly, increase the protection of the honey bees’ health. The modulation of the honey bee gut microbiota, by supplementation of selected LABs, has aroused special attention since it represents a strategy to improve the health status of colonies, in terms of productivity, as well as boosting the presence of beneficial microorganisms within the gut of new-generation bees.

Moreover, appropriate probiotics could be exploited to actively counteract bee pathogens and parasites, or to enhance immunity, and thus indirectly, increase the protection of the honey bees’ health.

However, further studies are necessary to gain a better mechanistic understanding of how host–microbe–environment interactions influence honey bee mortality and colony loss.

## Figures and Tables

**Table 1:** Overview of the main results obtained using LABs as probiotics in the honey bee diet.

LAB Species	Source	Relevant Reported Results	Ref.
*L. kunkeei* *F. fructosus*	Honey bee gastrointestinal tract	Decreases of the mortality rate and significant enhancement of the longevity of honey bees.	[64]
*L. johnsonii*		Queen egg-laying stimulation; higher number of honey bees and a significant increase in honey yield, healthier bee colony	[65,66,67]
*L. johnsonii* *L. kunkeei* *L. plantarum* *L. salivarius*		Increased honey production	[42,68]
*Bifidobacterium* spp. *Lactobacillus* spp.		Mild increment in bee survival	[69]
*B. lactis* *L. acidophilus* *L. casei*	Commercial probiotic product	Enhancement of bee health. Increased honey production and size of the wax cells	[70,71,72]
*B. bifidum* *L. acidophilus* *L. delbrueckii* sub. *bulgaricus*		Administration in pollen substitute resulted in an increase in dry mass and crude fat level	[73]
*L. brevis*		Increased expression of genes encoding antimicrobial peptides (abaecin, defensin-1)	[74]
*L. plantarum,* *L. rhamnosus*		Mitigate antibiotic-associated microbiota dysbiosis and immune deficits in adult workers	[75]
LAB mix: *B. breve* *B. longum* *L. acidophilus* *L. casei* *L. plantarum* *L. rhamnosus*	Commercial probiotic product	Enhance honey bee immunity. Higher levels for abaecin and defensin in honey bee larvae	[76]
Multiple LAB species		Bo0sting colonies’ strength. Positive physiological changes in probiotic-treated groups of adult bees	[77]
*B. bifidum* *E. faecium* *L. acidophilus* *P. acidilactici*		Advantages of probiotic supplementation include better bee survival and higher dry mass and crude fat level	[73]
*B. asteroids* *F. fructosus* *F. pseudoficulneus* *F. tropaeoli*	Honey bee hive	Induced immune stimulation (higher level of Apidaecin1). Results suggest that the bee immune response to endogenous bacteria is species-specific	[78]
*L. kunkeei*		Mitigate antibiotic-associated microbiota dysbiosis	[75]
*Fructobacillus* spp.		Able to utilize lignin and promote the growth of honey bee gut community members	[79]
*E. thailandicus* *L. curvatus* *W. cibaria* *W. viridescens* *W. paramesenteroides*	Different sources	The transcription levels of antimicrobial peptide genes, such as abaecin, defensin, and hymenoptaecin, were found to increase significantly	[80]

Taxonomic references: *Lactobacillus kunkeei: Apilactobacillus kunkeei; Lactobacillus plantarum: Lactiplantibacillus plantarum; Lactobacillus salivarius: Ligilactobacillus salivarius*.

**Table 2:** Overview of the main results obtained using LABs for the control of the honey bee diseases.

Disease	LAB Species	Source	Relevant Reported Results	Ref.
Chalkbrood	*Al. kunkeei* *L. plantarum*	Honey bee gastrointestinal tract	In vitro growth inhibition of *A. apis*	[154,176,177,178]
	*L. helsingborgensis* *Al. kunkeei* *L. melliventris*		Reduced in situ larval mummification by percentages greater than 80%.	[176,179]
American Foulbrood (AFB)	*Al. kunkeei* *L. brevis* *L. plantarum* *L. amylovorus* *L. fructivorans* *L. gasseri* *L. kunkeei* *L. apis*	Honey bee gastrointestinal tract	In vitro growth inhibition of *P. larvae*	[177,180,181,182]
	*B. asteroids* *B. coryneforme* *L. kunkeei* *and six Lactobacillus* spp.		Reduced honey bee larvae mortality.	[183,184]
American Foulbrood (AFB)	*B. asteroides* *B. coryneforme* *L. apinorum* *L. apis* *L. kunkeei* *L. kimbladii* *L. kullabergensis* *L. helsingborgensis* *L.* *mellis* *L.* *mellifer* *L.* *melliventris*	Honey bee gastrointestinal tract	The secretome of the LAB mixture strongly inhibited *P. larvae* vegetative growth.	[185]
	*L. kunkeei* *L. plantarum* *L. rhamnosus*	Honey bee hive	Reduced pathogen load and improved survival during *P. larvae* infection	[186,187]
	*Enterococcus* spp.		In vitro growth inhibition of *P. larvae* due to the production of bacteriocins.	[188]
	*E. durans*			[189,190]
	*E. thailandicus* *L. curvatus* *L. plantarum* *W. cibaria* *W. paramesenteroides* *W. viridescens*	Different sources	In vitro growth inhibition of *P. larvae*	[80,155]
	*Enterococcus* spp.		In vitro growth inhibition of *P. larvae* due to bacteriocin production	[191]
European foulbrood (EFB)	*L. apis*	Honey bee gastrointestinal tract	In vitro growth inhibition of *M. plutonius*.	[182]
	*L. kunkeei* and other thirteen LABs		Administration of LAB supplemented food in vivo and in vitro caused partial and total growth inhibition, respectively, and following decreases in the mortality rate	[192]
	*Al. kunkeei*		Purified bacteriocin (kunkecin A) exhibited high antibacterial activity against *M. plutonius*	[193]
	*L. kunkeei*	Honey	Antibacterial activity against *M. plutonius*	[46]
Nosemosis	*L. johnsonii*	Honey bee gastrointestinal tract	Reduction in *Nosema* spp. spores in hives	[66]
	*L. kunkeei*		Decreased the count of *N. ceranae* spores from adult honey bees.	[187]
	*L. salivarius*		Significant decrease in the spore levels of *Nosema* spp.	[194]
	*L. johnsonii*		In syrup administration of the metabolites produced by *L. johnsonii* (mainly organic acids), reduced the intensity of the disease	[67]
	*B. asteroides* *B. coryneforme* *B. indicum* *L. johnsonii* *L. kunkeei* *L. plantarum*	Honey bee hive	Reduction in *N. ceranae* spores	[195]
	*Enterococcus* spp.	Honey bee bread	The CFS (cell-free supernatant) does not affect spore viability as well as *N. ceranae* development	[196]
	*L. casei* *L. plantarum*	Commercial probiotic products	Higher mortality in honey bees treated with the probiotic formula, caused by an increase in the *Nosema* spp. infection	[197]
	*E. faecium*		Reduced *N. ceranae* proliferation and infected honey bees increased survivorship	[198]
	*L. rhamnosus*		Lower survival of honey bees fed with probiotic; rapid development of nosemosis in bees fed with the probiotic	[199]
	*P. acidilactici*		In vivo experimental infection by *N. ceranae*, showing a significant increase in survival rate (20–30%)	[200]
	Multiple species of LAB		Reduction in spore counts in colonies	[77]
	*P. acidilactici* *L. plantarum*		Regulate genes involved in honey bee development (vitellogenin), immunity (serine protease 40, defensin), and possibly prevent infection by the parasite *N. ceranae*	[131]
Varroosis	*L. salivarius*	Honey bee gastrointestinal tract	In situ reduction in the levels of varroosis between 50 and 80%	[194]
	*E. faecium* *L. johnsonii*		Enhance honey bee survival and increase their soluble proteins	[201]
	*B. asteroids* *L. kunkeei*	Surface of freshly collected bees	Caused 95–100% mortality of mites in 3 days	[202]

Taxonomic references: *Lactobacillus kunkeei: Apilactobacillus kunkeei; Lactobacillus plantarum: Lactiplantibacillus plantarum; Lactobacillus salivarius: Ligilactobacillus salivarius*.

## Data Availability

Not applicable.
